# Supplementary material for: Colloidal Crystal Films with Narrow Reflection Bands by Hot-Pressing of Polymer-Grafted Silica Particles
Source: Polymers (Basel). 2022 Nov 27;14(23):5157. doi: 10.3390/polym14235157 (PMC9736521; doi:10.3390/polym14235157)
Supplement: Supplementary file 1 [file polymers-14-05157-s001.zip › polymers-2069026-supplementary.pdf]

Submitted to *Polymers* as an Article

Supplementary Materials

Colloidal Crystal Films with Narrow Reflection Bands by Hot-Pressing of Polymer-Grafted Silica Particles

Sawa Matsuura, Mami Obara, Naoto Iwata* and Seiichi Furumi*

Department of Chemistry, Graduate School of Science,
Tokyo University of Science
1−3 Kagurazaka, Shinjuku, Tokyo 162−8601, Japan

*Correspondence: n-iwata@rs.tus.ac.jp (N.I.) and furumi@rs.tus.ac.jp (S.F.)

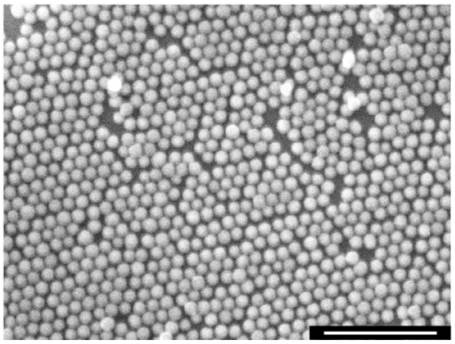

Figure S1. SEM image of the commercial silica particles (Seahostar KE-W10, Nippon Shokubai Co., Ltd) used in this study. The white scale bar represents 1 μm.

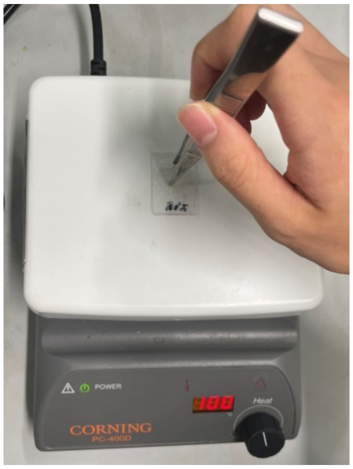

Figure S2. Photograph of hot-pressing by hand.

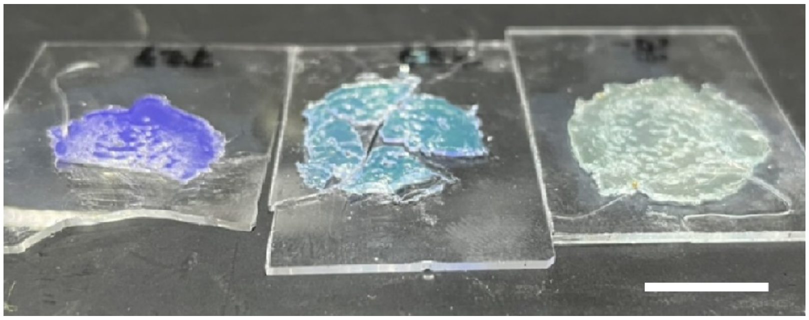

Figure S3. Reflection images of the CC films of SiP-POA109k (left side), SiP-POA191k (center) and SiP-POA278k (right side) observed at an oblique angle of ~45°. The white scale bar means 1 cm.

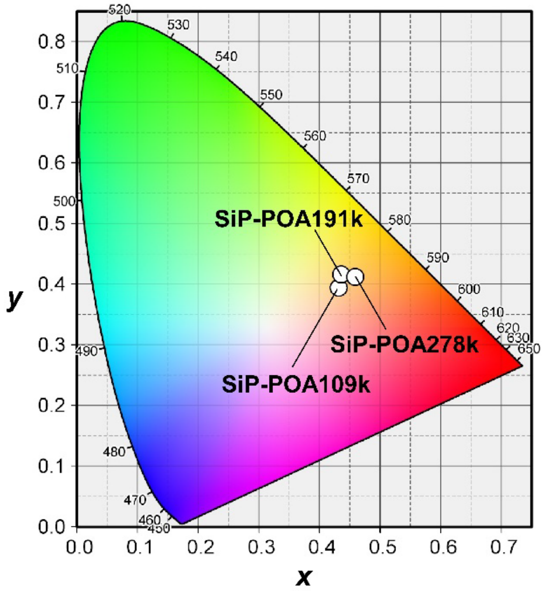

Figure S4. CIE coordinate values for the CC films of SiP-POA109k, SiP-POA191k and SiP-POA278k.
